# Abnormal overexpression of G9a in melanoma cells promotes cancer progression via upregulation of the Notch1 signaling pathway

**DOI:** 10.18632/aging.102750

**Published:** 2020-02-03

**Authors:** Ning-Ning Dang, Jing Jiao, Xianguang Meng, Yunhe An, Chen Han, Shuhong Huang

**Affiliations:** 1 Department of Dermatology, Jinan Central Hospital Affiliated to Shandong University, Jinan, Shandong Province, China; 2 Beijing Center for Physical and Chemical Analysis, Beijing, China; 3 Institute of Basic Medicine, The First Affiliated Hospital of Shandong First Medical University, Jinan, Shandong Province, China

**Keywords:** melanoma, G9a, UNC0642, Notch1, proliferation

## Abstract

Malignant melanoma is a type of very dangerous skin cancer. Histone modifiers usually become dysregulated during the process of carcinoma development, thus there is potential for a histone modifier inhibitor as a useful drug for cancer therapy. There is a multitude of evidence regarding the role of G9a, a histone methyltransferase (HMTase), in tumorigenesis. In this study, we first showed that G9a was significantly upregulated in melanoma patients. Using the TCGA database, we found a significantly higher expression of G9a in primary melanoma samples (n = 461) compared to normal skin samples (n = 551). Next, we knocked down G9a in human M14 and A375 melanoma cell lines *in vitro* via small interfering RNA (siRNA). This resulted in a significant decrease in cell viability, migration and invasion, and an increase in cell apoptosis. UNC0642 is a small molecule inhibitor of G9a that demonstrates minimal cell toxicity and good *in vivo* pharmacokinetic characteristics. We investigated the role of UNC0642 in melanoma cells, and detected its anti-cancer effects *in vitro* and *in vivo*. Next, we treated cells with UNC0642, and observed a significant decrease in cell viability in M14 and A375 cell lines. Furthermore, treatment with UNC0642 resulted in increased apoptosis. In immunocompetent mice bearing A375 engrafts, treatment with UNC0642 inhibited tumor growth. Results of Western blot analysis revealed that administration of UNC0642 or silencing of G9a expression by siRNA reduced Notch1 expression significantly and decreased the level of Hes1 in A375. All in all, the data from our study demonstrates potential of G9a as a therapeutic target in the treatment of melanoma.

## INTRODUCTION

Malignant melanoma, which develops from pigment-containing cells called melanocytes [1, 2], is one of the most aggressive forms of skin cancer. Melanomas are most commonly located in the skin, but can also occur in the mouth, intestines, or eyes. As the the most dangerous type of skin cancer [3], there were approximately 232,000 new occurrences of melanoma in 2012 across the world. In 2015, there were approximately 3.1 million people with active disease, resulting in 59,800 deaths [3–5]. Melanoma most commonly develops when people with low skin pigmentation levels are exposed to ultraviolet light. Therefore, wearing sunscreen and avoiding ultraviolet rays can contribute to the prevention of melanoma [4]. The primary treatment for melanoma is removal by surgery. When melanoma has spread, utilization of immunotherapy, biologic therapy, radiation therapy, or chemotherapy may improve survival, but resistance to conventional chemotherapy and radiotherapy is common. In the United States, there is a 98% five-year survival rate among patients with a localized tumor, but only a 16% five-year survival rate once the melanoma has spread [6].

G9a functions as a histone methyltransferase (HMTase), modulating the methylation of histones H3K9 and H3K27 [7, 8]. H3K9 methylation controlled by G9a is an essential process of transcriptional repression of many related genes during cancer development [9–14]. There has been an increasing amount of evidence regarding the role of G9a in tumorigenesis [15, 16]. G9a is unregulated in various cancers, including human bladder [17, 18], lung [19–21], colon [9, 22] and breast cancer [14, 23–26], compared with normal tissue. G9a also contributes to the inhibition of E-cadherin expression in ovarian and endometrial cancer [27–29]. Therefore, a valuable therapeutic target could be focused on the decreased expression of G9a expression. G9a has been reported to be a potential prognostic marker in patients with melanoma [30]. However, the tumorigenic role of G9a in melanoma is largely unknown.

In this study, we observed dysregulation of G9a in melanoma samples, repression of Notch1 and Hes1 expression via G9a inhibitor UNC0642, and inhibition of melanoma cell growth. We were able to demonstrate that Notch1 regulation contributes to the tumorigenic activity of G9a, which suggests a new function of G9a in controlling tumor growth.

## RESULTS

### G9a is upregulated in melanoma and its expression demonstrates a positive correlation with cancer progression

**Figure 1:**
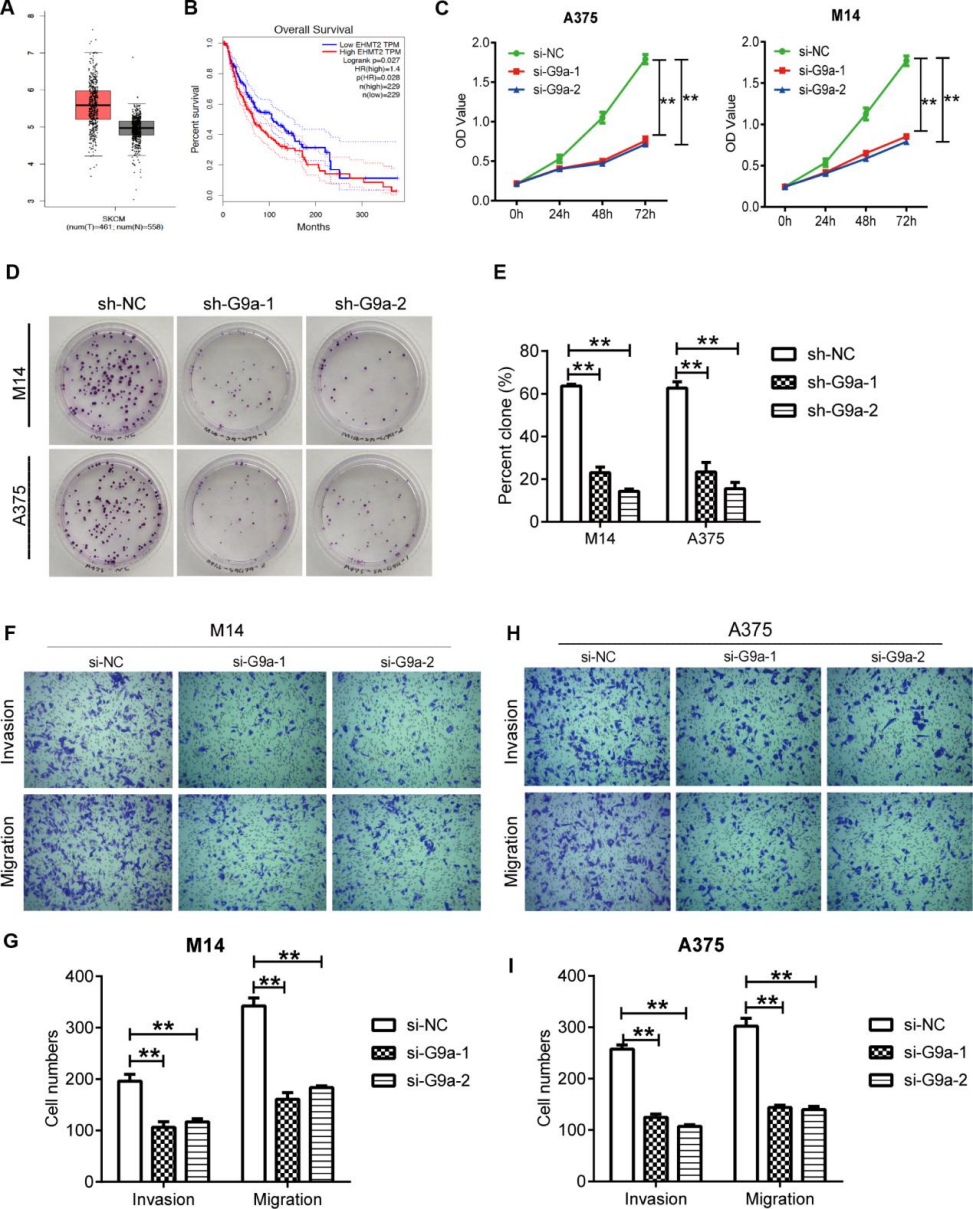
**Knockdown of G9a inhibited the proliferation and metastasis of human melanoma cells.** (**A**) the mRNA expression of G9a was analyzed via GEPIA. The red and grey boxes represent Skin Cutaneous Melanoma (SKCM) and normal skin samples, respectively. (**B**) the prognostic value of G9a (EHMT2 equivalent to G9a) was analyzed via GEPIA. (**C**) CCK8 was performed to detect the proliferation of M14 and A375 cells transfected with two G9a specific-siRNAs (si-G9a-1 and si-G9a-2 group) or negative control siRNA (si-NC group). (**D**) cell proliferation was further confirmed by colony formation assay. (**E**) percent clone was analyzed for M14 and A375 cells. (**F**) transwell was performed to detect the migration and invasion of M14 cells. (**G**) the number of migratory and invasive cells was counted for M14 cells. (**H**) transwell was performed to detect the migration and invasion of A375 cells. (**I**) the number of migratory and invasive cells was counted for A375 cells. ** P<0.01.

To investigate the role of G9a in melanoma development, we analyzed the normalized level of mRNA expression (TPM) of G9a in melanoma patients using the GEPIA database and its online analysis tool. As shown in Figure 1A, there was a relative higher average of mRNA expression of G9a in primary melanoma samples (n = 461) compared to normal skin samples (n = 558; P < 0.05; Figure 1A). The Kaplan-Meier curve indicated that G9a was closely correlated with poorer survival outcomes (log-rank test, P=0.006, Figure 1B).

Dysfunction of cell proliferation, migration and apoptosis are crucial events that may promote cancer development. Therefore, analysis of the role of the target gene on typical cellular behavior of cancer cells is important for studying certain gene functions. So, we sought to elucidate the cellular behaviors of melanoma cells under the control of G9a. First, CCK assay was utilized to detect the effect of G9a on the proliferation of M14 cells. As shown in Figure 1C, the results indicate a remarkable decrease in the rate of cell proliferation of si-G9a groups (si-G9a-1 and siG9a-2) compared to the si-NC group, suggesting an effective role of G9a on cell proliferation. We further used clone formation assay to investigate cell proliferation of M14 and A375 cells. To get a prolonged knockdown effect, we constructed the stable knockdown cell lines using lenti-virus carrying sh-G9a-1 and 2 sequences. As shown in Figure 1D, the number of clones in both of the sh-G9a group decreased to approximately 40% of that of the siNC group in both cell lines (Figure 1D, 1E). All of the results indicated that G9a played a crucial role in maintaining melanoma proliferation. We further examined the migration and invasion ability of the cells upon both of si-G9a transfection by transwell. As shown, there was a significant decrease in the migration and invasion ability of siG9a group cells compared with si-NC group cells (Figure 1F–1I). These results suggest that G9a promotes the migration and invasion of melanoma cell lines.

### G9a regulated migration and proliferation of melanoma cells through EMT-related and PI3K-AKT pathways

**Figure 2:**
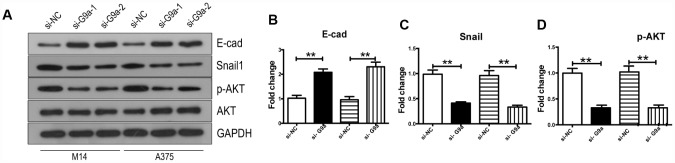
**Knockdown of G9a reversed EMT and inhibited the activity of AKT in human melanoma cells.** (**A**) Western blot was used to detect the expression levels of E-Cad, Snail, AKT and p-AKT. (**B**) the column diagram showed the protein level of E-Cad. (**C**) the column diagram showed the protein level of Snail. (**D**) the column diagram showed the protein level of p-AKT. ** P<0.01.

Next, we investigated potential relevant signaling pathways to determine the detailed mechanisms by which G9a promotes melanoma progression through Western blot analysis. The PI3K-AKT pathway is a frequently activated and dysregulated pathway in the development of cancers [32–35]. This pathway is involved in proliferation, apoptosis and migration of many cancer types, and abnormal activation of the AKT pathway has been reported in melanoma [36–39]. Therefore, we hypothesized that G9a may modulate the AKT pathway and consequently affects melanoma progression. So, we detected the phosphorylation levels of AKT in order to elucidate the downstream signal molecules of G9a in melanoma. As shown in Figure 2A–2D, there was a decrease in the levels of phosphorylated AKT without affecting the overall level of total AKT in siG9a groups. We further detected EMT related protein(s) by Western blot analysis. E-Cad expression was up-regulated in si-G9a groups, while there was a decrease in expression of the most important EMT-related transcription factor, Snail-1. According to the above results, G9a played a role in the activation of the AKT pathway and up-regulated E-Cad to promote proliferation and migration.

### G9a expression promoted Notch signaling activity in melanoma cells

**Figure 3:**
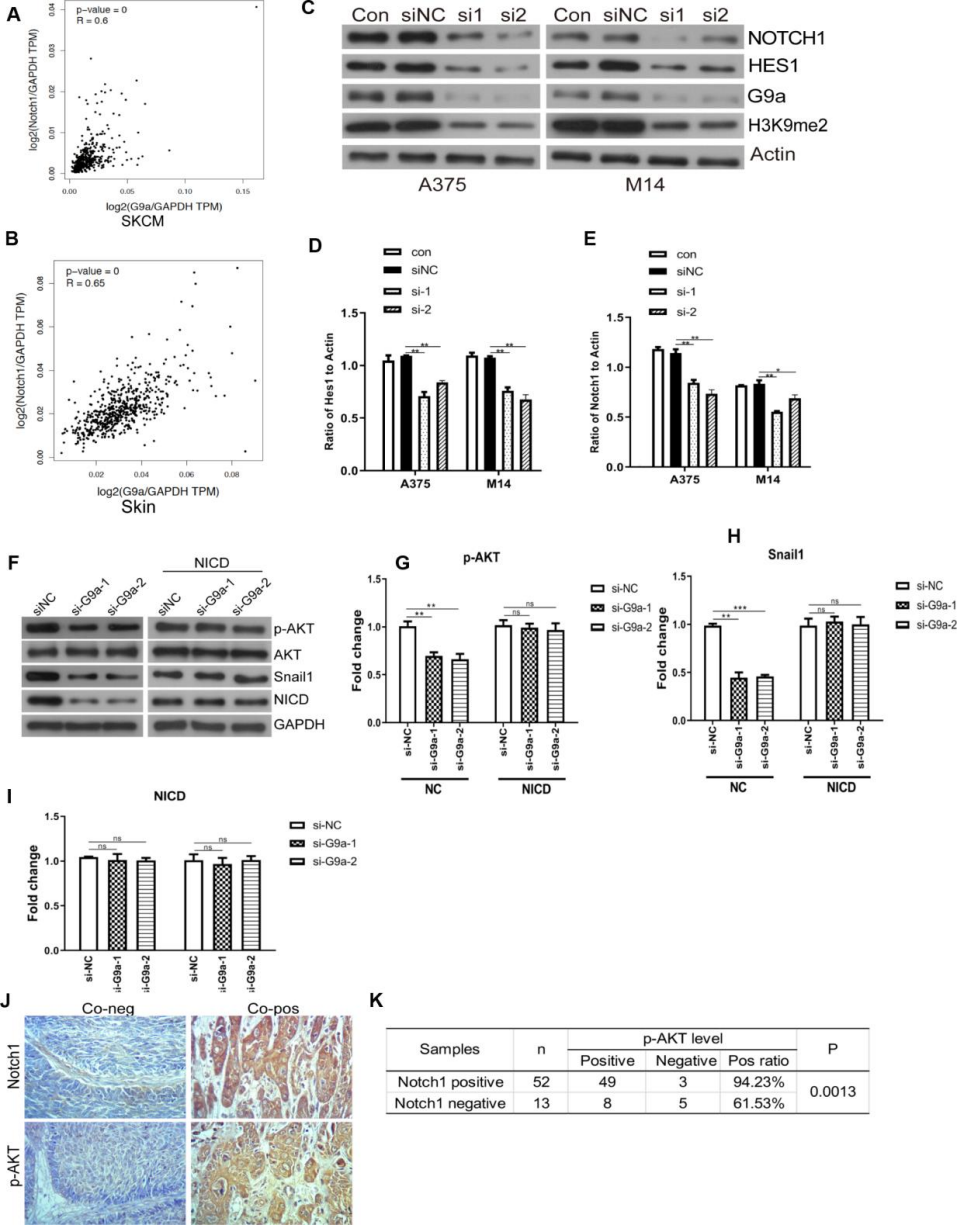
**G9a inhibited the Notch1 pathway in melanoma and normal skin cells.** (**A**) the correlation of G9a and Notch1 expression in SKCM samples was analyzed via GEPIA. (**B**) the correlation of G9a and Notch1 expression in normal skin samples was analyzed via GEPIA. (**C**) Western blot was performed to detect the expression of Notch1, Hes1 and H3K9me2 in G9a knockdown cells. (**D**) the column diagram showed the expression level of Hes1. (**E**) the column diagram showed the expression level of Notch1. (**F**) NICD was overexpressed in G9a knockdown cells (NICD group), and to detect the expression levels of p-AKT, Snail1 and NICD were detected by western blot. (**G**) the column diagram showed the expression level of p-AKT. (**H**) the column diagram showed the expression level of Snail1. (**I**) the column diagram showed the expression level of NICD. (**J**) IHC was performed to detect the expression of Notch1 and p-AKT in skin cancer tissues. (**K**) analysis of IHC showed the relationship between the expressions of Notch1 and p-AKT in human skin cancer. * P<0.05; ** P<0.01.

Notch signaling is a critical process for the malignancy of melanoma cells [40, 41]. In this study, we further investigated the role of G9a on Notch1 expression and activity in melanoma cells. We analyzed the relationships between different genes via GEPIA. As shown in Figure 3A and 3B, there was a moderate positive correlation between G9a and Notch1 (R=0.6, p<0.01; Pearson correlation analysis). Results from data analysis suggested potential interactions between G9a and Notch1 during melanoma development. This provided us with a direction for further research. We utilized Western blot analysis to detect whether G9a is involved in Notch1 activity in melanoma cell lines M14 and A375. As shown in Figure 3C–3F, along with the important substrate H3K9me2, the protein levels of Notch1 and Hes1 were also decreased in siG9a groups. To further address whether the role of G9a on PI3K-AKT signaling is dependent on Notch activity, we detected the phosphor-AKT and Snail1 by WB after ectopic expression of NICD (the active form of Notch1) and G9a knockdown. As shown in Figure 3G–3J, ectopic expression of NICD blocked the response for si-G9a. Further, the protein level of Notch 1 and p-AKT in melanoma samples was detected by IHC. As shown in Figure 3K and 3L. the p-AKT positive rate in Notch1-positive tissue was 94.23%, much higher than the rate in Notch1-negative tissue (61.53%). According to the above results, we can conclude that G9a activated the Notch1 pathway, and then, Notch1 signaling pathway further modulates the activity of PI3K-AKT.

### Inhibiting G9a with UNC0642 reduced Notch signaling activity in melanoma cells lines

**Figure 4:**
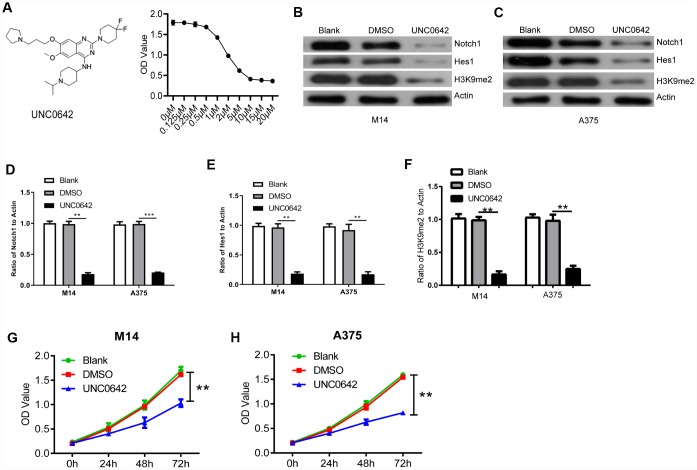
**UNC0642 inhibited the Notch1 signaling pathway.** (**A**) the structural formula of UNC0642 (left panel), and the IC50 value was calculated via a dose dependent assay (right panel). (**B**) the expression of Notch1, Hes1 and H3K9me2 in M14 cells treated with UNC0642 or DMSO was detected with M14 cells as a blank control. (**C**) the expression of Notch1, Hes1 and H3K9me2 in A375 cells was detected using Western blot. (**D**) the column diagram showed the related expression of Notch1 in A375 and M14 cells. (**E**) the column diagram showed the related expression of Hes1 in A375 and M14 cells. (**F**) the column diagram showed the related expression of H3K9me2 in A375 and M14 cells. (**G**) CCK8 assay was performed to detect the proliferation of M14 cells treated with UNC0642 or DMSO. (**H**) CCK8 assay was performed to detect the proliferation of A375 cells treated with UNC0642 or DMSO. ** P<0.01; *** P<0.001.

UNC0642 is a newly discovered inhibitor of G9a, which demonstrates high cellular potency and excellent selectivity in numerous cell lines [18] For instance, we used it for further targeting of G9a of melanoma cells both *in vitro* and *in vivo*. In order to identify whether UNC0642 affects the cellular functions of melanoma cells, different concentrations of UNC0642 were used to treat melanoma cell lines A375 and detected by CCK8 assay. Our results showed that UNC0642 had no effect on survival of A375 cells at the concentration lower than 0.5nM. At the concentration of 2 μM or higher, UNC0642 had a significant inhibitory effect on the viability of A375 cells in a dose-dependent manner (Figure 4A). The IC50 of UNC0642 was 3.062 μM for A375 cells, and 2 μM of UNC0642 was used to treat M14 and A375 cells for the rest experiments (Figure 4A). Further we detected the role of UNC0642 on the activity of Notch1 signaling by WB. As shown in Figure 4B–4F, the protein levels of Notch1, Hes1 and H3K9me2 decreased in UNC0642 groups. Further we performed CCK8 assay to investigate the effect of G9a on the proliferation of melanoma cells. Results from the CCK8 assay revealed that UNC0642 treatment significantly reduced the proliferation of M14 and A375 cells (Figure 4G–4H).

### Ectopic expression of NICD can rescue the inhibition of cellular viability, migration and invasion upon UNC0642 or siG9a treatment

**Figure 5:**
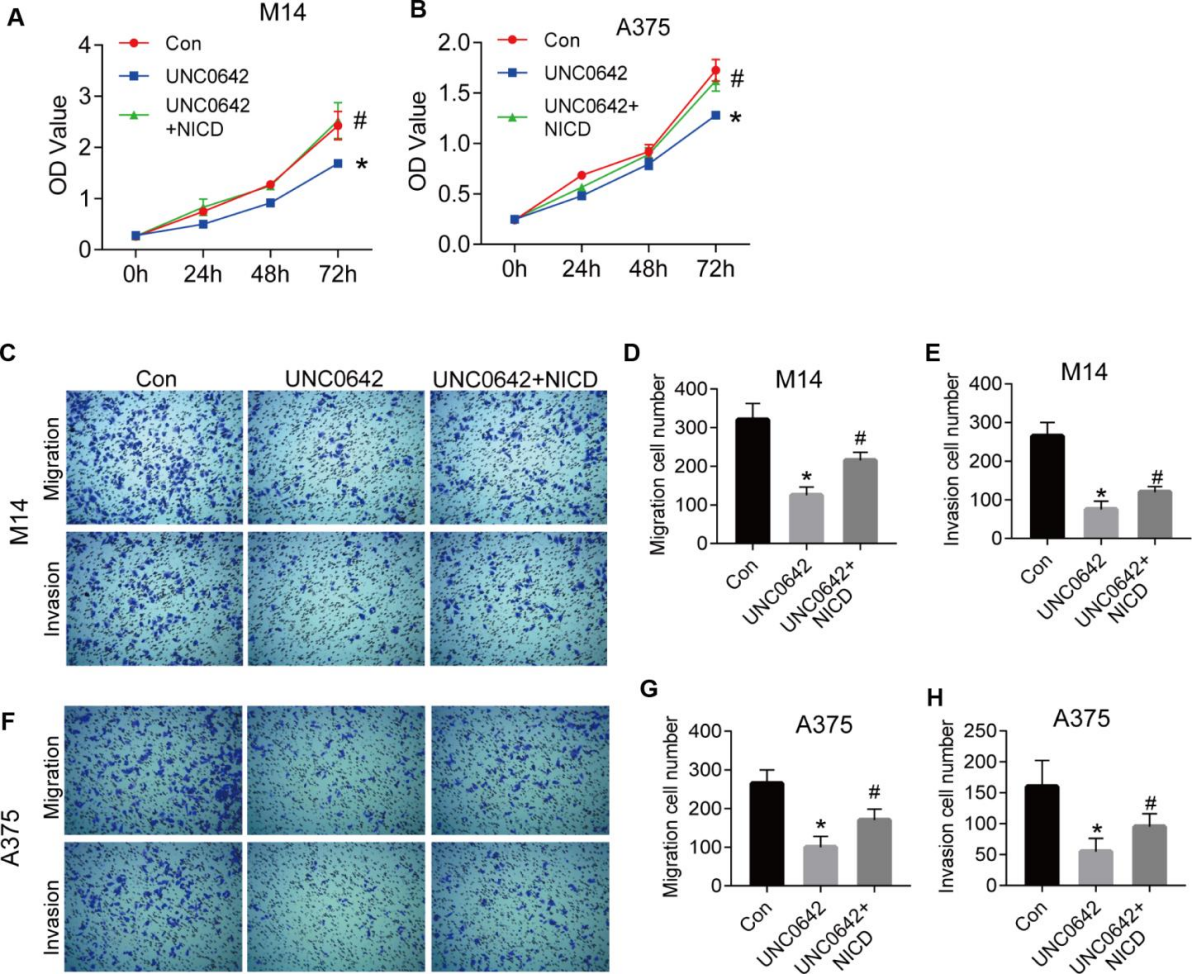
**NICD blocked the inhibition of proliferation and metastasis induced by UNC0642 in human melanoma cells.** (**A**) the proliferation of M14 cells with the treatment of UNC0642 or both UNC0642 and NICD was detected by CCK8. (**B**) the proliferation of A375 cells with the treatment of UNC0642 or both UNC0642 and NICD was detected by CCK8. (**C**) the migration and invasion of M14 cells with the treatment of UNC0642 or both UNC0642 and NICD was detected by transwell. (**D**) the column diagram showed the migration cell number in M14 cells. (**E**) the column diagram showed the invasion cell number in M14 cells. (**F**) the migration and invasion of A375 cells with the treatment of UNC0642 or both UNC0642 and NICD was detected by transwell. (**G**) the column diagram showed the migration cell number in A375 cells. (**H**) the column diagram showed the invasion cell number in A375 cells. *P<0.05 vs. Con group; #P<0.05 vs. UNC0642 group.

To explore whether G9a modulates cell viability via the Notch1 pathway in melanoma cells, the CCK-8 assay was utilized to evaluate the viability of M14 and A375 cell lines with UNC0642. Results from the CCK8 assay revealed that UNC0642 treatment significantly reduced the proliferation of M14 and A375 cells, but cell viability was restored after ectopic expression of NICD, the active form of Notch1 (Figure 5A, 5B). We next evaluated the effect of UNC0642 on cell migration and invasion via transwell assay. The transwell assay analysis showed that UNC0642 treatment resulted in significantly reduced migration and invasion of M14 and A375 cells, whereas both migration and invasion in M14 and A375 cells were restored to some extent after overexpression of NICD (Figure 5C–5H). In sum, these data indicate that G9a can regulate melanoma cell viability, migration and invasion via the Notch1 signaling pathway.

**Figure 6:**
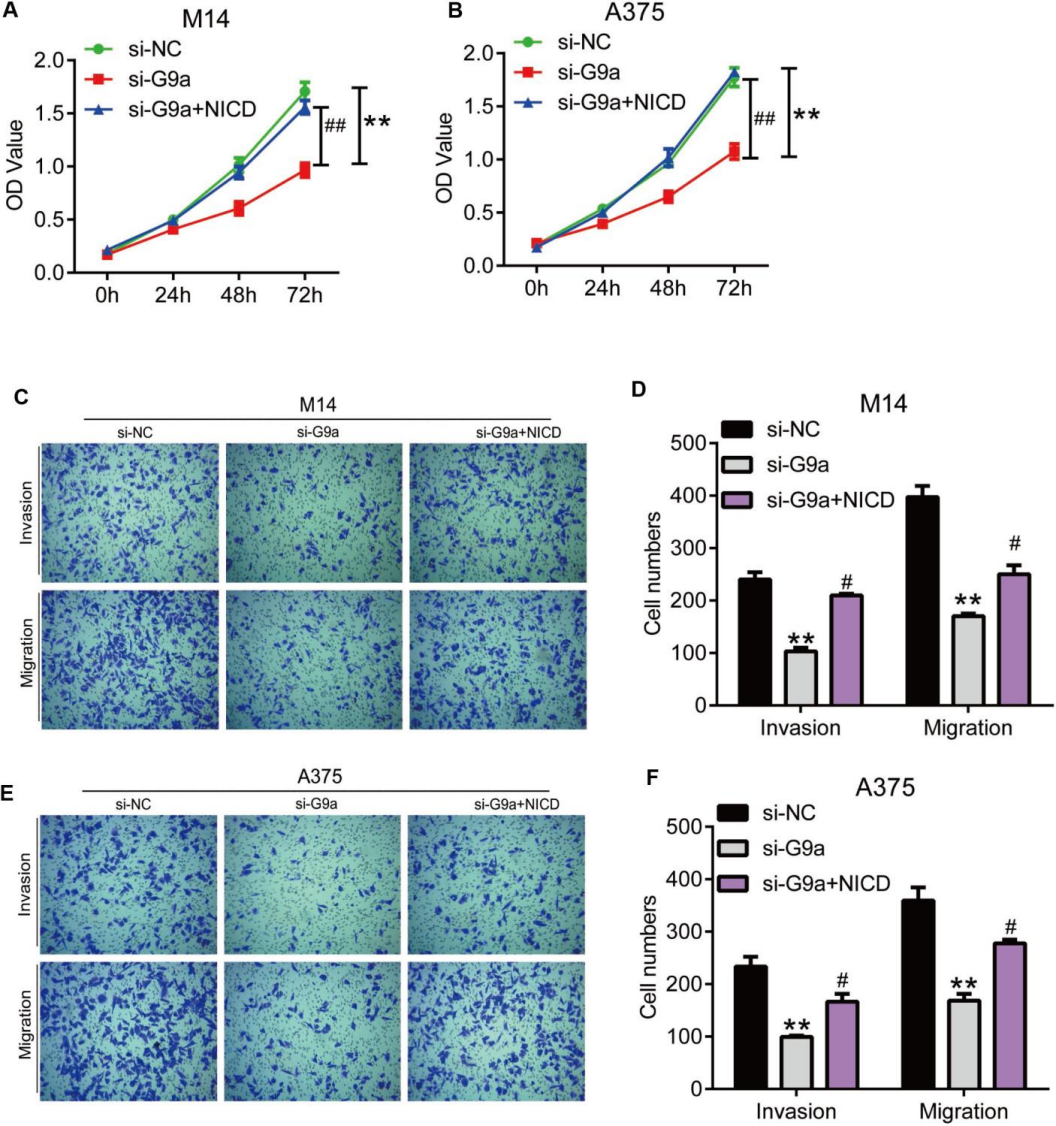
**NICD blocked the inhibition of proliferation and metastasis induced by G9a in human melanoma cells.** (**A**) the proliferation of M14 cells with G9a knockdown or both G9a knockdown and NICD treatment was detected by CCK8. (**B**) the proliferation of A375 cells with G9a knockdown or both G9a knockdown and NICD treatment was detected by CCK8. (**C**) the migration and invasion of M14 cells with G9a knockdown or both G9a knockdown and NICD treatment was detected by transwell. (**D**) the column diagram showed the migration and invasion cell number in M14 cells. (**E**) the migration and invasion of A375 cells with G9a knockdown or both G9a knockdown and NICD treatment was detected by transwell. (**F**) the column diagram showed the migration and invasion cell number in A375 cells. **P<0.01 vs. Con group; #P<0.05 vs. si-G9a group.

To exclude the potential off-target of UNC0642, we confirmed the above experiments with G9a knocking down by siRNA (Figure 6). As shown in Figure 6A and 6B, the CCK8 assay revealed that siG9a treatment significantly reduced the proliferation of M14 and A375 cells, but cell viability was restored after ectopic expression of NICD. The transwell assay analysis showed that siG9a transfection resulted in significantly reduced migration and invasion of M14 and A375 cells, whereas both migration and invasion in M14 and A375 cells were restored to some extent after overexpression of NICD (Figure 6C–6F).

### Ectopic expression of NICD can inhibit the apoptosis induced by UNC0642 treatment

**Figure 7:**
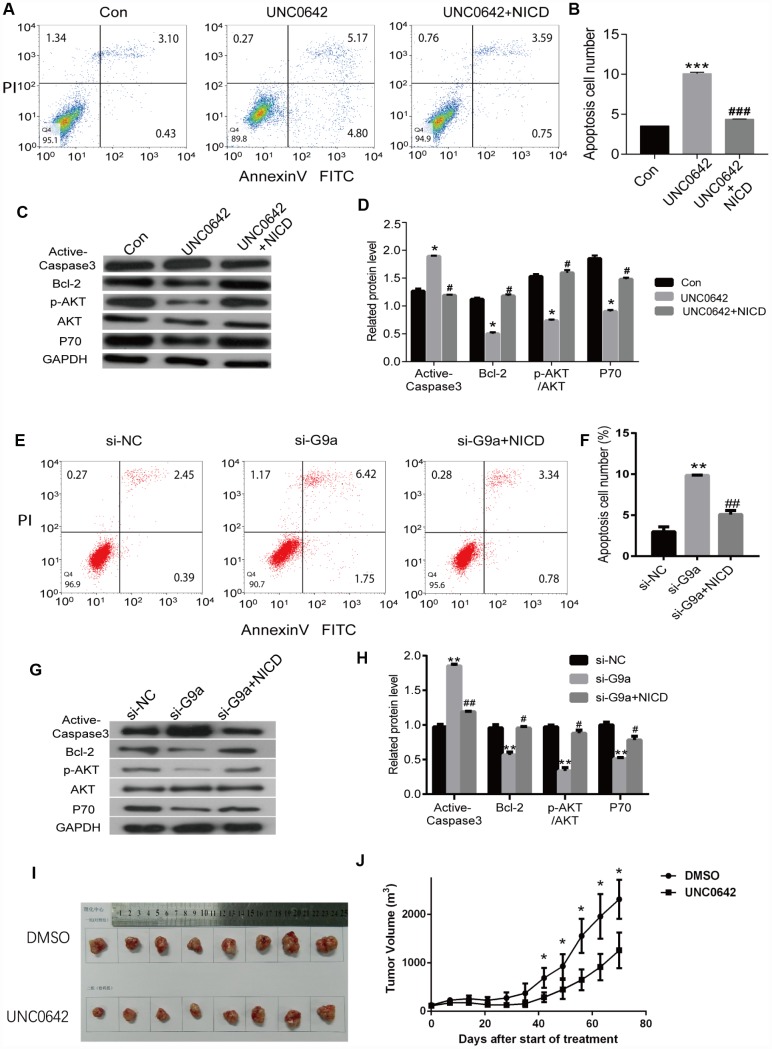
**NICD inhibits UNC0642-induced apoptotic enhancement in melanoma cells.** (**A**) the apoptosis of melanoma cells with the treatment of UNC0642 or both UNC0642 and NICD was detected by flow cytometry. (**B**) the column diagram showed the apoptosis cell number. (**C**) the apoptosis related protein levels were detected by western blot in melanoma cells with the treatment of UNC0642 or both UNC0642 and NICD. (**D**) the column diagram showed the related expression levels of target genes. (**E**) the apoptosis of melanoma cells with G9a knockdown or both G9a knockdown and NICD treatment was detected by flow cytometry. (**F**) the column diagram showed the apoptosis cell number. (**G**) the apoptosis related protein levels were detected by western blot in melanoma cells with G9a knockdown or both G9a knockdown and NICD treatment. (**H**) the column diagram showed the related expression levels of target genes. (**I**) the nude mice were inoculated with melanoma cells with the treatment of DMSO or UNC0642. (**J**) the tumor volume was measured. *P<0.05, **P<0.01 and *** P<0.001 vs. Con group; #P<0.05, ##P<0.01 and ###P<0.001 vs. UNC0642 group.

To evaluate whether UNC0642 is capable of inducing apoptosis, we double stained melanoma cells treated with UNC0642 with Annexin V-FITC/PI. Using flow cytometry analysis, we observed an increase in the ratio of apoptotic cells upon UNC0642 treatment, and the ectopic expression of NICD could inhibit this apoptosis caused by UNC0642 treatment (Figure 7A, 7B). Moreover, there was a significant increase in the expression of apoptosis marker, cleaved Caspase-3, in melanoma cells upon UNC0642 treatment according to Western blot analysis, while the expression of Bcl-2 was remarkably reduced (Figure 7C, 7D). Meanwhile, we sought to determine whether UNC0642 treatment had an effect on the PI3K-AKT signaling pathway. The results revealed a decrease in the activity of the PI3K-AKT signaling pathway in melanoma cells treated with UNC0642 at concentrations of 2 μM (Figure 7C, 7D). Ectopic expression of NICD could rescue above results (Figure 7A–7D). Further, we confirmed the above experiments with G9a knocking down by siRNA. As shown in Figure 7E, 7F, we observed an increase in the ratio of apoptotic cells upon siG9a transfection, and the ectopic expression of NICD could inhibit this apoptosis caused by siG9a transfection. At the same time, the expression of cleaved Caspase-3, in melanoma cells upon siG9a transfection increased significantly according to Western blot analysis, while the expression of Bcl-2 was remarkably reduced (Figure 7E–7H). And ectopic expression of NICD could be used to restore values. These results indicate that inhibiting G9a with UNC0642 induces apoptosis through the Notch1 signaling pathway in melanoma cells.

### Inhibiting G9a with UNC0642 suppressed tumor growth *in vivo*

To evaluate the *in vivo* effects of G9a inhibition, nude mice received subcutaneous injections of A375 cells. One week after xenografts became palpable, UNC0642 (5 mg/kg) was administered via i.p. injection every other day for 10 days. During the treatment window, UNC0642 inhibited tumor growth (P < 0.05; Figure 7I, 7J) without significantly altering body weight compared with the control (data not shown). Thus, UNC0642-mediated targeting of G9a was able to suppress melanoma tumor growth *in vivo*.

## DISCUSSION

The abnormal expression of G9a has been investigated in multiple cancer types [15]. In breast or gastric carcinoma cells [14, 23, 24], silencing of G9a via RNA interference can inhibit cell proliferation and invasion, suggesting that G9a overexpression is critical for cancer cell survival and aggression. We observed an upregulation of G9a in human melanoma patients. Moreover, overexpression of G9a showed a significant correlation with poor prognosis in melanoma patients from the TCGA Provisional dataset.

The aberrant upregulation of G9a in melanoma suggests it is a good candidate as a potential therapeutic molecular target. We transfected G9a siRNA into M14 and A375 cells and investigated the change of biological phenomenon. The results showed that silencing of G9a reduced cell proliferation, migration, and invasion in human melanoma cells, and also induced apoptosis. In addition, the results showed that si-G9a caused downregulation of BCL-2 and significant upregulation of cleaved-Caspase 3, which are both involved in apoptosis. Studies have shown that p-AKT activates or inhibits its downstream target proteins Caspase3, P70 and NFκB through phosphorylation, thereby regulating cell proliferation, apoptosis and migration. In this study, G9a regulated the expression of Caspase3 and P70 by activating AKT pathway, and then regulated the proliferation and apoptosis of melanoma cells.

Although G9a seems promising as a drug target, many small molecule inhibitors have been identified with significant *in vitro* inhibitory effects against G9a activity [16]. A specific inhibitor of G9a, UNC0642, is an effective and specific inhibitor of G9a with high *in vitro* cellular potency, perfect selectivity and greatly improved *in vivo* pharmacokinetic properties [42–44]. Inhibition of G9a by UNC0642 induces apoptosis of human bladder cancer cells [18]. In this study, UNC0642 was applied to G9a, which resulted in decreased cell viability and invasion, and caused apoptosis in melanoma cell lines. Finally, our *in vivo* data showed that UNC0642 suppressed the tumorigenicity of melanoma xenografts.

The Notch family receptors are involved in regulating the development, differentiation, and cellular function of multiple cell types [45–49]. In particular, the Notch1 signaling pathway has demonstrated a close relationship with melanoma progression [40, 41, 50–55]. For example, studies have proven that Notch1 signaling promotes the progression of primary melanoma through activation of mitogen-activated protein kinase/phosphatidylinositol 3-kinase-Akt pathways and upregulation of N-cadherin expression [56]. However, the relationship between G9a and Notch1 is largely unknown. In addition, we found that there was a significant correlation between the expression of Notch1 and p-AKT in skin cancer tissues by using IHC. Studies have shown that the expression of Notch 1 and p-AKT has significant correlation in a variety of tumors, and share the same downstream molecules. In melanoma cells, hyperactivated PI3K/Akt signaling led to upregulation of Notch1 through NF-kappa B activity [57]. In another research, inhibition of Notch1 induce anti-melanoma effects via activating both the MAPK and PI3K/Akt pathways [58]. All the above results suggest that there is a cross effect between Notch 1 and Akt signaling pathway, which can indirectly induce tumor formation.

In the present study, we further investigated the role of G9a expression in melanoma cells on the Notch1 signaling pathway. We found that depletion of G9a reduces the activity Notch1 signaling pathway activity, and the ectopic expression of NICD rescues the inhibition of cellular viability, migration and invasion due to UNC0642 treatment.

The mechanisms underlying Notch1 transcriptional regulation via G9a should also be discussed. According to previous reports, G9a activity increases, causing an increase in global histone methylation [15], and further cause transcriptional repression of different tumor suppressors, including CDH1, DUSP5, SPRY4, and RUNX3 [15]. Apart from histones, G9a could also methylate other proteins, such as another chromatin-remodeling factor-Pontin [59], p53 [60] and MyoD [61]. While the implications of this mechanism are not fully understood. So further investigation still needed to uncover the mechanism of G9a dependent Notch1 regulation. Given UNC0642 mainly play roles on HMT activity inhibition, G9a might cause transcriptional repression of certain regulator of Notch1, and subsequently down-regulated Notch1.

In conclusion, this study was not only helpful for elucidating the role of G9a in melanoma, but also provided data regarding the relationship between G9a and Notch1 signaling in melanoma cells. Our study is the first to report that G9a regulates melanoma cell function, and that targeting of G9a by UNC0642 significantly inhibits melanoma cell proliferation and survival *in vitro* and *in vivo*. All in all, we were able to demonstrate the potential of UNC0642 as a therapeutic option for melanoma patients.

## MATERIALS AND METHODS

### Chemicals and antibodies

UNC0642 (#U4885) was purchased from Sigma (St. Louis, MO, USA). The antibodies specific for EHMT2/G9A (1:2000; ab185050), β-actin (1:5000; ab179467) and glyceraldehyde 3-phosphate dehydrogenase (GAPDH) (1:5000; ab181602) were purchased from Abcam Inc. The other antibodies included Notch1 antibody (1:1000; ab194123, Abcam) and Hes1 antibody (1:1,000; ab221788, Abcam).

### Cell culture

Commercialized melanoma cell lines M14 and A375 were purchased from the American Type Culture Collection (Manassas, VA, USA). Cells were cultured in high-glucose Dulbecco’s modified Eagle’s medium (Invitrogen), then supplemented with 10% fetal bovine serum (Invitrogen) and 100 U ml−1 penicillin and 100 μg ml−1 streptomycin. Cross-contamination with other human cells was evaluated, and all cells tested negative. siRNA and overexpression plasmid were transfected using Lipofectamine RNAiMax (#13778500) and Lipofectamine 2000 (#11668019, Invitrogen, Carlsbad, CA, USA), respectively, according to the manufacturer’s instructions. The target sequence of siRNA were as follow: si-G9a-1: 5’-CTCACTGACAACGAGGAGAACATCTGCCT-3’ and si-G9a-2: 5‘-CGTTACTATGGCAACATCAGCCGCTTCAT-3’.

### Real-time reverse-transcription PCR

TRIzol (#15596018, Invitrogen) was utilized to isolate total cellular RNA, which was reverse transcribed into complementary DNA using the PrimeScript RT reagent Kit ((Invitrogen, Carlsbad, CA, USA). An Applied Biosystems 7500 apparatus was utilized to perform real-time reverse-transcription PCR using the SYBR-Green Master mix (#RR820B, Takara). The following primers were used: G9a 5ʹ-GCCAGGCCGGGAGGCCCTGGAA-3ʹ (sense), 5ʹ-CTCCAGCCTGCAGCAGCACATG-3ʹ (antisense); Notch1 5ʹ-ATGCACTGCCATGTGACTGA-3ʹ (sense), 5’-CTTGGTGATGACGGTGAGG-3ʹ (antisense); and GAPDH 5ʹ-GCAAATTCCATGGCACCGTC-3ʹ (sense), 5ʹ-TCGCCCCACTTGATTTTG-3ʹ (antisense). The experiment was performed at 96 °C for 10 min, followed by 45 cycles at 95 °C for 5 s, and 61 °C for 20 s. Triplicate assays were performed for all samples. The comparative Ct method was utilized to calculate the fold change in RNA, providing normalization to the GAPDH control.

### Colony formation assay and cell proliferation assay

M14 and A375 knockdown cells were seeded into six-well plates at a density of 1000 cells per well, then cultured for 14 days until the appearance of colonies was evident. The colonies were fixed in 10% formaldehyde at room temperature for 15 min, followed by staining with 1% Crystal Violet. CCK8 was utilized for the cell proliferation assay. Normal cultured M14 and A375 cells were trypsinized and counted to create the suspension. About 1000 cells were seeded into each well of the 96-well plate, and cells treated with 0.1% DMSO were used as the control. A total of 10 μl of CCK8 reagent was added every 24 hours to detect cell vitality. Following 90 minutes of incubation at 37 °C, the OD value of excitation light was detected using enzyme standard instrument at a wavelength of 450 nm. Based on the OD values, a proliferation curve was drawn.

### Flow cytometry

M14 and A375 cells were stained with Annexin V–fluorescein isothiocyanate (FITC) and propidium iodide (PI). Flow cytometry was utilized for the detection of cell apoptosis, according to the manufacturer’s protocol. Following treatment, the cells were collected via centrifugation. The cell pellets obtained from centrifugation were suspended in 500 μl of binding buffer, then 5 μl of Annexin V-FITC and 5 μl of a PI solution were applied prior to incubation at room temperature for 15 min. A FACSCalibur instrument was used to measure Annexin V and PI staining by flow cytometry, and data analysis was performed via FlowJo software.

### Cell transwell and invasion assays

Prior to beginning the experiment, the coating buffer (consisting of 0.01M Tris pH 8.0, 0.7% NaCl and filtered by 0.2 μm sterile filter unit) was prepared. Any pipets, syringes, or containers that could come into contact with the Matrigel were chilled prior to use. Overnight, the Matrigel Matrix aliquot was thawed on ice at 4 °C, then it was diluted with serum-free 1640 medium at a ratio of 1:6. A total of 100 μl was applied to each permeable support well of the 24-well plate prior to incubation at 37 °C for 4 hours. Without disturbing the layer of Matrigel, we carefully removed the permeable support membrane and added 100 ul and 600 ul of serum-free 1640 medium, respectively, to the inside and outside. Next, we incubated the membrane at 37 °C for 30 minutes. At this point, the coated invasion chambers were ready for use. The procedure for the transwell experiment was similar to the invasion experiment, but a cubicle wasn’t required for Matrigel processing, and the number of cells was 5000.

### Immunohistochemistry (IHC)

Skin cancer tissue Microarray was purchased from Shanghai Outdo Biotech Co.,Ltd. (Shanghai, China).

Protein expression was detected using IHC and analyzed based on previous descriptions [31]. The representative pictures were taken with a microscope (OPTIKA B-150, ITALY). Image Pro Plus6.0 (Media Cybernetics) was used for the protein expression analysis of five random fields each sample.

### *In vivo* xenograft study

Six-week old male nude mice (Male C57BL/6 and BALB/c Nude mice) received subcutaneous injections of A375 cells (2X 106). After one week, nude mice with palpable xenografts were randomly assigned to two groups. Vehicle (DMSO) was used as treatment for one group, while UNC0642 (5 mg/kg) was used for the other group via intraperitoneal (i.p.) injection every other day. A caliper was used to measure tumor volumes, which were calculated as length X width^2^/2. At the end of the experiment, the mice were euthanized, and the tumors were weighed and processed for further analysis.

### Statistical analysis

Statistical analysis was performed via SPSS 18.0 software, and the results were denoted as plus or minus standard deviation (SD). This is indicated in the legend of the figure. The data was obtained from three separate experiments. Student’s t-test was used to compare pairs of interest. P<0.05 was considered statistically significant.
